# Contributions of External, Muscle, and Ligament Forces to Tibiofemoral Contact Loads in Patients with Knee Osteoarthritis and Healthy Individuals

**DOI:** 10.3390/bioengineering12060600

**Published:** 2025-05-31

**Authors:** Li Zhang, Peng Xu, Hui Li, Chao Lu, Weikun Hou, Aibin Zhu, Pingping Wei

**Affiliations:** 1 Honghui Hospital, Xi’an Jiaotong University, Xi’an 710054, China; xjtuzhangli@xjtu.edu.cn (L.Z.); drlihuiortho@gmail.com (H.L.); weikunhou@163.com (W.H.); 2 Shaanxi Key Laboratory of Intelligent Robots, Institute of Robotics and Intelligent Systems, Xi’an Jiaotong University, Xi’an 710049, China; abzhu@mail.xjtu.edu.cn; 3 State Key Laboratory for Manufacturing Systems Engineering, Xi’an Jiaotong University, Xi’an 710054, China; erin.wei@mail.xjtu.edu.cn

**Keywords:** knee biomechanics, knee osteoarthritis, musculoskeletal modeling, ligament forces, muscle forces, external forces, gait analysis

## Abstract

This study aims to quantify the contributions of external, muscle, and ligament forces to the tibiofemoral contact loads during gait. Additionally, the relative contributions in patients with knee osteoarthritis (KOA) and healthy individuals were also compared. For this aim, twenty medial Kellgren–Lawrence (KL) 3–4 KOA patients and twenty healthy controls were recruited to perform the gait data collection experiment using a motion capture and force plate system. The relative contributions were calculated based on an improved musculoskeletal model with knee ligaments. The results showed that the contribution of muscle forces to the total compartment contact loads was greater than that of external forces for both the healthy individuals and the KOA patients. The medial compartment contact loads were contributed predominantly by external forces, and the lateral compartment contact loads were contributed negatively by external forces for both the healthy individuals and the KOA patients. For the healthy individuals, the total/lateral compartment contact loads were predominantly contributed by muscle forces. The ligament forces provide a contribution similar to muscle forces to the medial compartment contact loads. For the KOA patients, the total/lateral compartment contact loads were contributed predominantly by ligament forces. The ligament forces provide a negative contribution to the medial compartment contact loads. In conclusion, the knee ligaments provided important contributions to the tibiofemoral contact loads. Significant differences were found in the relative contributions between the KOA patients and the healthy individuals. The results of this study have significant clinical implications for further improving the current biomechanical treatments of KOA.

## 1. Introduction

Knee osteoarthritis (KOA), as one of the common degenerative diseases characterized by chronic pain and decreased mobility, seriously affects the quality of life of older adults and causes significant economic burdens to patients, families, and society [1,2]. An epidemiological study shows that the prevalence of KOA in Asia, Europe, and North America is 19.2%, 13.4%, and 15.8%, respectively, with approximately 650 million people affected worldwide [3]. Changes in tibiofemoral compartment contact loads caused by aging and obesity play a significant role in the development and progression of KOA [4].

Tibiofemoral compartment contact loads are the combined mechanical loads of external, surrounding muscles, surrounding ligaments, and other soft tissue forces [4,5]. External forces not only support the body weight but alter the load distribution between knee medial and lateral compartments by external knee adduction moment (EKAM) [6,7]. The forces generated by muscles, ligaments, and other soft tissue forces are aimed at stabilizing the tibiofemoral joint to resist external forces. However, these forces will increase the contact loads on the tibiofemoral compartment [8,9]. The current biomechanical treatments of KOA are mainly to reduce the tibiofemoral compartment contact loads. Therefore, the biomechanical treatments of KOA should comprehensively consider the effect of external forces, as well as muscle, ligament, and other soft tissue forces [4].

For patients with early or mid-stage KOA who are not suitable for knee replacement surgery, biomechanical treatments can postpone the progression of KOA and have been widely used clinically in recent years [10]. The knee brace based on the three-point bending theory is targeted to apply an orthopedic moment in the knee frontal plane to reduce the EKAM [11]. The wedge insole alters the EKAM by reducing the lever arm from the ground reaction force to the knee center [12]. The gait modifications reduce the EKAM by toeing in, toeing out, increasing trunk sway, medial knee thrust, etc. [13]. Additionally, the treatment of weight loss is intended to reduce the total external forces on the knee joint, although it has little effect on the EKAM [10]. The above biomechanical treatments can primarily reduce the contributions of external forces on the tibiofemoral compartment contact loads. The purpose of strength training is to increase the surrounding muscles’ strength [10]. The powered knee assistive device and kinesio taping aim to provide a supplement to the surrounding muscle forces [14,15]. These treatments can improve the stability of the knee joint, thereby altering the contribution of muscle forces on the tibiofemoral compartment contact loads. To some extent, the improvements of joint alignment and biomechanical environment of the knee joint by using the above biomechanical treatments can change the passive forces of surrounding ligaments and then alter the contribution of ligament force on the tibiofemoral compartment contact loads [16]. Therefore, it is important to understand the contributions of external, muscle, and ligament forces to the tibiofemoral compartment contact loads in order to further improve the effect of the current biomechanical treatments.

Due to the invasiveness and cost, direct in vivo measurements of the tibiofemoral compartment contact loads, surrounding muscle forces, and surrounding ligament forces are unfeasible. Musculoskeletal modeling provides a viable approach to compute the relative contributions of each part to the tibiofemoral compartment contact loads. Winby et al. [17] investigated the contributions of external and muscle forces to the tibiofemoral compartment contact loads during normal gait in healthy subjects. They found that the muscle forces were the major contributors to the total, medial, and lateral compartment contact loads. Similar to the study of Winby et al. [17], Konrath et al. [18] focused on patients with anterior cruciate ligament (ACL) reconstruction using semitendinosus and gracilis tendon (ST + GR) grafts. They studied the contributions of muscles and external forces to the medial compartment contact loads during normal walking, running, and cutting. The results showed that the medial compartment contact loads were contributed predominantly by the external forces during normal walking and by the muscle forces during running and cutting. However, the target subjects of these two studies were not KOA patients. The two studies did not analyze the contributions of ligament forces due to the use of musculoskeletal models without knee ligaments. Brandon et al. [19] analyzed the contributions of muscles and external forces to medial compartment contact loads in patients with moderate medial KOA. They observed that the major contributor to the medial compartment contact loads was the external forces. However, the study did not investigate the contributions to the total and lateral compartment contact loads. A musculoskeletal model without knee ligaments was still utilized in their study. After adding the knee ligaments model to the musculoskeletal model, Shelburne et al. [20] investigated the contributions of muscles, ligaments, and external forces to the tibiofemoral compartment contact loads during normal gait in healthy individuals. They found that muscle forces were the major contributors to the total or lateral compartment contact loads, and external forces were the major contributors to the medial compartment contact loads. Additionally, the negative contribution of external forces that acted to unload the lateral compartment was also found in their study. However, the musculoskeletal model utilized in their study was a single lower limb model with thirteen muscles and eight ligaments, which was relatively simple compared to the musculoskeletal models in the studies of Winby et al. [17], Konrath et al. [18], and Brandon et al. [19]. In addition, the research subjects of their study were young healthy males rather than KOA patients and older healthy individuals. In conclusion, although the research mentioned above analyzed the relative contributions of each part to the tibiofemoral compartment contact loads, no previous studies have analyzed the relative contributions of external, muscle, and ligament forces to the tibiofemoral compartment contact loads for KOA patients and compared the relative contributions between KOA patients and healthy individuals.

Therefore, the purpose of this study was to quantify the contributions of external, muscle, and ligament forces to the tibiofemoral contact loads on the total, medial, and lateral compartments during gait based on an improved musculoskeletal model with knee ligaments. The relative contributions of KOA patients and healthy individuals were compared. We hypothesized that the knee ligaments provided an important contribution to the tibiofemoral contact loads. KOA would alter the relative contributions of external, muscle, and ligament forces to the tibiofemoral contact loads on the total, medial, and lateral compartments.

## 2. Materials and Methods

### 2.1. Participants

The protocol received ethical approval through the Ethics Committee of our hospital (registration number: 202309006 and data of approval: 9 October 2023). All participants provided their written informed consent. The purpose and all procedures of our study were fully explained to each participant prior to any testing. All methods of our study were performed in accordance with the Declaration of Helsinki.

In order to reduce the possibility of adverse events during the experiment and to reduce confounding variables, participants were excluded if they could not walk independently without assistive devices, had lower limb surgeries or injuries within the last two years, or had any significant rheumatoid, neuromuscular, metabolic or cardiovascular diseases. Twenty patients with symptomatic and radiographic KOA on the medial compartment of both knees (Kellgren–Lawrence (KL) grades: 3–4 [21]), diagnosed by an orthopedic surgeon at our hospital, were recruited as the KOA patients group. Twenty age-matched healthy participants with no history of any lower limb disease were recruited as the healthy control group. Demographic information for the two groups is presented in Table 1. For the KOA patients group, eight knee joints had KL grade 3, and thirty-two knee joints had KL grade 4. The average duration of KOA symptoms was 13.7 ± 6.9 years.

Data from this study were from a secondary analysis in our previous study about the knee biomechanical characteristics of KOA patients and healthy individuals [22]. The power (1-β) for this study was estimated by using the G*Power software (version 3.1.9.7, University of Dusseldorf, Düsseldorf, Germany). Using the effect size from the previous study published by Winby et al. [17], the sample size of 20 KOA patients and 20 controls, and an α level of 0.05, the power (1-β) for this study was computed to be 0.95. Thus, the current sample size yielded sufficient power for the statistical approach.

### 2.2. Experimental Instruments

**Figure 1:**
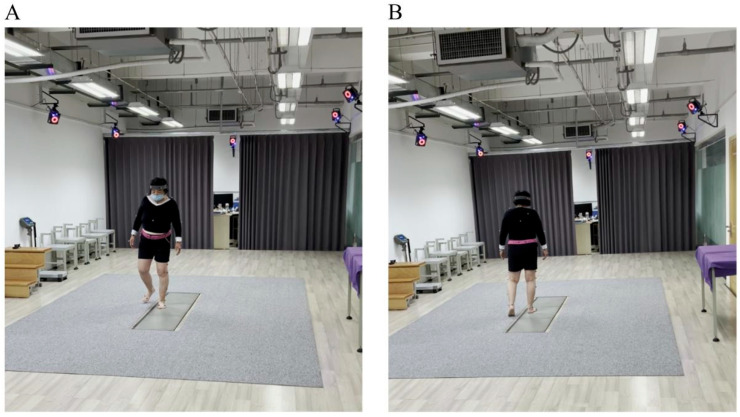
Gait data collection experiment: (**A**) gait trail for left limb, (**B**) gait trail for right limb.

Concurrent and synchronous motion capture and force plate systems were utilized to capture raw gait data in this study (see Figure 1). Thirty-seven retro-reflective skin-surface markers (15 mm) were adhered to the full body of each participant in accordance with previously published marker settings [22]. A 10-camera VICON MX motion analysis system (Vicon version 3.3, Vicon Motion Analysis Inc., Oxford, UK) was used to capture the 3-dimensional (3D) motion of all makers with a sampling frequency of 100 Hz. The 3D ground reaction forces (GRF) were obtained from three 200 × 300 mm^2^ force plates (BP 600600, Advanced Mechanical Technology Inc., Watertown, MA, USA) embedded in the laboratory floor sampling at 1000 Hz. Motion capture and force plate systems are connected to the MX data conversion console through the MX connection line and then connected to the PC for concurrent and synchronous data capture. The Vicon Nexus software (version 2.6.1, Vicon Motion Analysis Inc., Oxford, UK) was used for the data collection.

### 2.3. Experimental Procedure

Gait task was performed at the 3D Gait Analysis Laboratory of our university. Participants were familiarized with the gait task about 30 min prior to data collection. Then, the participants were instructed to walk barefoot at self-selected speed along 15 m walkway with force plates. Due to the stride length of our recruited participants being relatively small compared to the length of the force plate (300 mm), the participants were instructed to walk with one foot on the force plate and the other foot on the floor (see Figure 1). Therefore, two kinds of gait trails were used for each participant: one for the right limb and one for the left limb. For each participant and each kind of trail, at least ten successful trails were used. A trial was considered successful if the relevant foot landed wholly on the force plate.

Subsequently, standing full-length radiography was performed by one radiographer for each participant to measure the femur length, tibia length, and pelvis width and determine the contact points of the knee joint [22,23].

### 2.4. Musculoskeletal Model

A new musculoskeletal model based on a previously published model in AnyBody Modeling System (version 7.4, AnyBody Technology, Aalborg, Denmark) was proposed in this study [22]. The previously published musculoskeletal model could calculate the tibiofemoral contact loads on the total, medial, and lateral compartments. The model had two improvements compared to the generic model in AnyBody Modeling System [24]. Firstly, the knee joint and ankle joint were updated to 3-DOF joints. Secondly, the knee joint was updated to a medial/lateral two-point contact joint. The contact points were identified by the standing full-length radiography and weighted center of proximity algorithm [23].

By adding the knee ligaments, the model could better predict tibiofemoral contact loads, which has been validated in the studies of Vanheule et al. [25], Marra et al. [26], Hu et al. [27], and Zhang et al. [28]. To improve the predicted accuracy and compute the contributions of external, muscle, and ligament forces to the tibiofemoral contact loads, we added ten knee ligaments (22 bundles) to the previously published model. The ligaments we modeled included the anterior/posterior cruciate ligaments (ACL/PCL, 2/2 bundles), medial/lateral collateral ligaments (MCL/LCL, 5/3 bundles), medial/lateral patellofemoral ligaments (MPFL/LPFL, 3/3 bundles), anterolateral ligament (ALL, 1 bundle), posteromedial ligament (PML, 1 bundle), posterior oblique ligament (POL, 1 bundle), and popliteofibular ligament (PFL, 1 bundle). For the geometrical properties, the effect of ligament-bone contact was neglected, and the path of each bundle was considered as a straight line. The attachment sites of each bundle were estimated according to the description found in the literature [29,30]. For the mechanical properties, the ligament bundle was assumed to be a piecewise nonlinear-elastic bundle, which meant that the bundle force (F_b_) was a function of its strain (*ε*) [28].

(1) $$\begin{array}{c} F_{b}=\left\{\begin{array}{cc} k(\varepsilon -\varepsilon_{lim}) & \varepsilon >2\varepsilon_{lim}\\ 1/4\varepsilon^{2}/\varepsilon_{lim} & 0\leq \varepsilon \leq 2\varepsilon_{lim}\\ 0 & \varepsilon <0 \end{array}\right. \end{array}$$

where *ε_lim_* is the reference linear strain limit and is set at 0.03 [25]. Two mechanical parameters of each ligament bundle, the stiffness (*k*) and strain in knee full extension (*ε_r_*), were obtained from the published studies [25,26,31]. In these studies, the *k* and *ε_r_* were measured through in vitro tensile testing of the human cadaveric knee ligaments. The specific values of *k* and *ε_r_* for each ligament could be found in our previous published study [32].

### 2.5. Experimental Data Processing

The raw C3D file output by the Vicon Nexus software (version 2.6.1) included the raw maker trajectories and 3D GRF from each subject’s gait experiment. We imported the raw C3D file into the musculoskeletal model we built in the AnyBody Modeling System for gait simulation analysis. The simulation process could be divided into parameter identification, inverse kinematics, and inverse dynamics. Firstly, parameter identification was performed to scale the musculoskeletal model to each participant using the marker trajectories and radiographic femur length, tibia length, and pelvis width. Secondly, inverse kinematics was utilized to calculate the joint angles. Finally, inverse dynamics was performed to calculate the total medial and lateral compartment contact loads, EKAM, muscle forces, ligament forces, net knee joint forces, etc. According to our previous study [22], the contact load along the longitudinal axis of the tibia was the main component of the tibiofemoral contact load and was much greater than the contact load in the other two directions. Thus, only the contact load along the longitudinal axis of the tibia was analyzed in this study. After the AnyBody simulation analysis, the contribution of external, muscle, and ligament forces to the total, medial, and lateral compartment contact forces was quantified by using the self-programmed programs based on the quasi-static frontal-plane moment balance in Python software (version 3.4.3, Python Software Foundation, Amsterdam, The Netherlands).

### 2.6. Statistical Analysis

All curves of contributions were normalized from 0% to 100% of the gait stance phase (heel strike to toe off). All data were presented as mean ± standard deviation (SD). The Shapiro–Wilk test was employed to test the normal distribution of all data. Demographic information of the KOA patients group and the healthy control group were compared at baseline using independent t-tests and chi-square tests. As shown in Table 1, there were no significant differences in demographic information such as age (*p* = 0.510), height (*p* = 0.259), weight (*p* = 0.621), BMI (*p* = 0.138), and sex (*p* = 0.715) between the KOA patients and the healthy individuals. Since the self-selected walking speed was different between the KOA patients group and the healthy control group (*p* < 0.001), we used a linear regression model to determine whether the self-selected walking speed was linearly related to the contributions of external, muscle, and ligament forces for each group. The results showed that there was no linear relationship between the walking speed and the contributions for the KOA patients group (*r*^2^ < 0.01, *p* = 0.531) or the healthy control group (*r*^2^ < 0.01, *p* = 0.476). Therefore, neither demographic information nor walking speed were included as covariates. The contributions of external, muscle, and ligament forces to tibiofemoral contact loads in both KOA patients and healthy individuals were compared using the independent t-test for parametric data and the Wilcoxon Signed-rank test for non-parametric data. The independent t-test and Wilcoxon Signed-rank test were followed with false discovery rate (FDR) corrections to adjust for multiple comparisons. The *p* value was adjusted using the Benjamini–Hochberg procedure. A *p* value less than 0.05 was considered as statistically significant, and all statistical analyses were performed using SPSS software (version 19, Chicago, IL, USA).

## 3. Results

**Figure 2:**
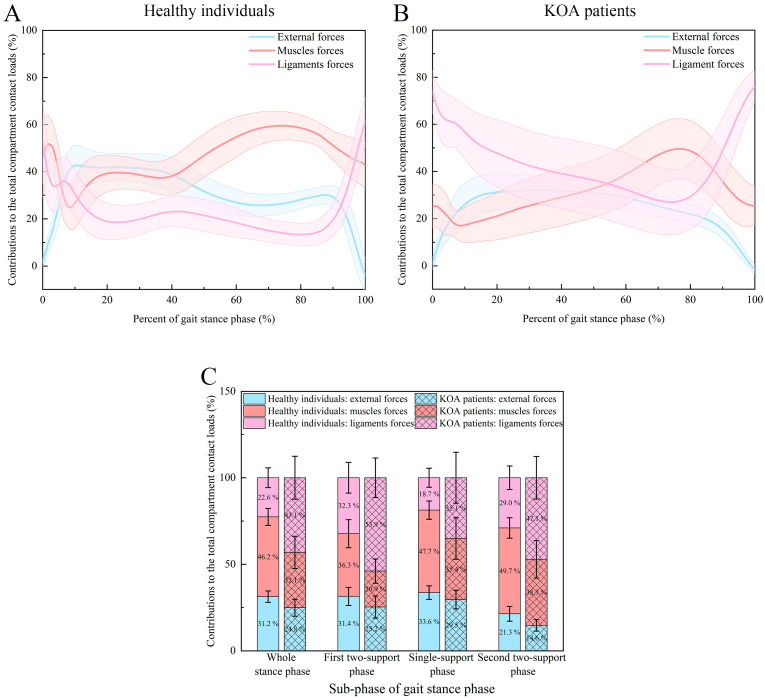
The contributions of external, muscle, and ligament forces to the total compartment contact loads: (**A**) contribution–time curves (mean: solid line, standard deviation: shaded area) for the healthy individuals (normalized from 0% to 100% of the gait stance phase); (**B**) contribution–time curves (mean: solid line, standard deviation: shaded area) for the KOA patients (normalized from 0% to 100% of the gait stance phase); (**C**) normalized contributions for the healthy individuals and the KOA patients during the whole stance phase, the first two-support phase, the single-support phase, and the second two-support phase.

The contributions of external, muscle, and ligament forces to the total compartment contact loads during the stance phase of the gait cycle are presented in Figure 2 and Table 2. For the healthy subjects, the total compartment contact loads were contributed predominantly by the muscle forces (46.2 ± 4.9%) followed by the external forces (31.2 ± 3.3%) and finally, the ligament forces (22.6 ± 5.7%) during the whole stance phase (*p* < 0.001). However, the KOA patients were different in that the ligaments and muscle forces were the primary and secondary contributors to the total compartment contact loads during the whole stance phase (43.1 ± 12.4% and 32.1 ± 9.3%), respectively, with the external forces contributing the remainder (24.8 ± 4.9%) (*p* < 0.001). For both the KOA patients and the healthy individuals, the contribution of muscle forces to the total compartment contact loads throughout the stance phase was larger than that of external forces (*p* < 0.001). During the first two-support phase, the muscle forces were the major contributors to the total compartment contact loads for the healthy individuals (36.3 ± 8.1%), whereas the ligament forces were the major contributors for the KOA patients (53.9 ± 11.4%) (*p* < 0.001). For the remaining contributions, the external and ligament forces had similar contributions for the healthy individuals (31.4 ± 5.3% and 32.3 ± 8.9%) (*p* = 0.601), but the external forces had greater contribution than the muscle forces for the KOA patients (25.2 ± 6.4% and 20.9 ± 7.0%) (*p* = 0.0191). During the single-support phase, the total compartment contact loads of the healthy individuals were still contributed predominantly by the muscle forces (47.7 ± 5.3%) (*p* < 0.001). But the contribution of external forces increased to 33.6 ± 4.0% (*p* = 0.0426), and the contribution of ligament forces decreased to 18.7 ± 5.5% (*p* < 0.001) compared to the first two-support phase. For the KOA patients, the muscle and ligament forces had similar major contributions throughout the single-support phase (35.4 ± 12.0% and 35.1 ± 14.7%) (*p* = 0.934), with the external forces contributing the remainder (29.5 ± 5.4%), unlike the first two-support phase. During the second two-support phase, the muscle forces and ligament forces were the main contributors to the total compartment contact loads for the healthy individuals (49.7 ± 5.9%) and the KOA patients (47.1 ± 12.3%), respectively (*p* < 0.001), like the first two-support phase and the whole stance phase. Compared to the single-support phase, the ligament forces increased the contributions to the total compartment contact loads, whereas the external forces decreased the contribution for both the KOA patients (47.1 ± 12.3% and 14.6 ± 3.3%) and the healthy individuals (29.0 ± 6.8% and 21.3 ± 4.3%) (*p* < 0.001). Furthermore, compared to the healthy individuals, the contribution of ligament forces was increased, and the contributions of muscles and external forces were decreased for the KOA patients during all of the sub-stance phases (*p* < 0.001).

**Figure 3:**
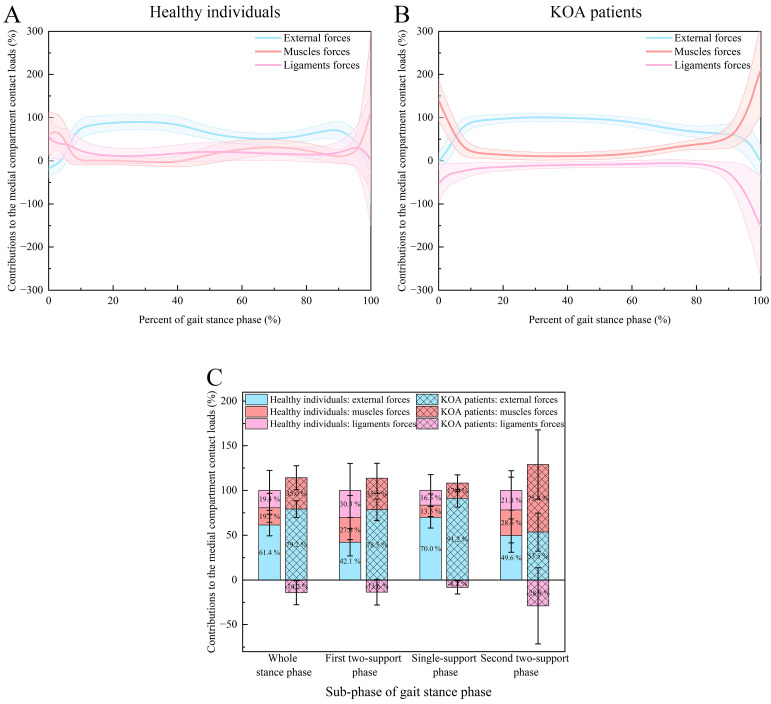
The contributions of external, muscle, and ligament forces to the medial compartment contact loads: (**A**) contribution–time curves (mean: solid line, standard deviation: shaded area) for the healthy individuals (normalized from 0% to 100% of the gait stance phase); (**B**) contribution–time curves (mean: solid line, standard deviation: shaded area) for the KOA patients (normalized from 0% to 100% of the gait stance phase); (**C**) normalized contributions for the healthy individuals and the KOA patients during the whole stance phase, the first two-support phase, the single-support phase, and the second two-support phase.

Figure 3 and Table 3 show the contributions of external, muscle, and ligament forces to the medial compartment contact loads during the stance phase of the gait cycle. For the healthy subjects, the external forces were the primary contributors to the medial compartment contact loads (whole stance phase: 61.4 ± 12.1%; first two-support phase: 42.1 ± 15.2%; single-support phase: 70.0 ± 12.0%; second two-support phase: 49.6 ± 18.6%) (*p* < 0.001). For the remaining contributions, there were no significant differences between the contributions of muscle and ligament forces for both the whole stance phase (19.2 ± 16.1% and 19.4 ± 22.3%, *p* = 0.747), the first two-support phase (27.6 ± 24.6% and 30.3 ± 30.2%, *p* = 0.563), the single-support phase (13.5 ± 12.6% and 16.5 ± 17.7%, *p* = 0.415) and the second two-support phase (28.6 ± 36.7% and 21.8 ± 22.0%, *p* = 0.467). Compared to the two-support phases, the contribution of external forces was significantly increased, but the contributions of muscle and ligament forces were significantly decreased during the single-support phase (*p* < 0.05). However, the contributions of external, muscle, and ligament forces had no significant differences between the first and second two-support phases (*p* = 0.112, 0.891, and 0.272). For the KOA patients, the medial compartment contact loads were still contributed predominantly by the external forces and followed by the muscle forces for both the whole stance phase (79.2 ± 9.3% and 35.0 ± 13.4%), the first two-support phase (78.5 ± 12.0% and 35.1 ± 16.8%) and the single-support phase (91.2 ± 9.7% and 17.0 ± 9.2%), whereas predominantly by the muscle forces and followed by the external forces for the second two-support phase (75.4 ± 38.7% and 53.5 ± 21.2%) (*p* < 0.001). It is remarkably, however, that the ligament forces of the KOA patients generated negative contributions to the medial compartment contact loads (whole stance phase: −14.2 ± 13.4%; first two-support phase: −13.6 ± 14.3%; single-support phase: −8.2 ± 7.4%; second two-support phase: −28.9 ± 42.6%). Like the healthy individuals, the external forces increased their contribution, while the muscle and ligament forces decreased their contributions during the single-support phase compared to the two-support phases for the KOA patients (*p* < 0.001). In general, the contributions of external forces and muscle forces were further increased, and the contribution of ligament forces was reduced to the negative contribution for the KOA patients compared to the healthy individuals (*p* < 0.001).

**Figure 4:**
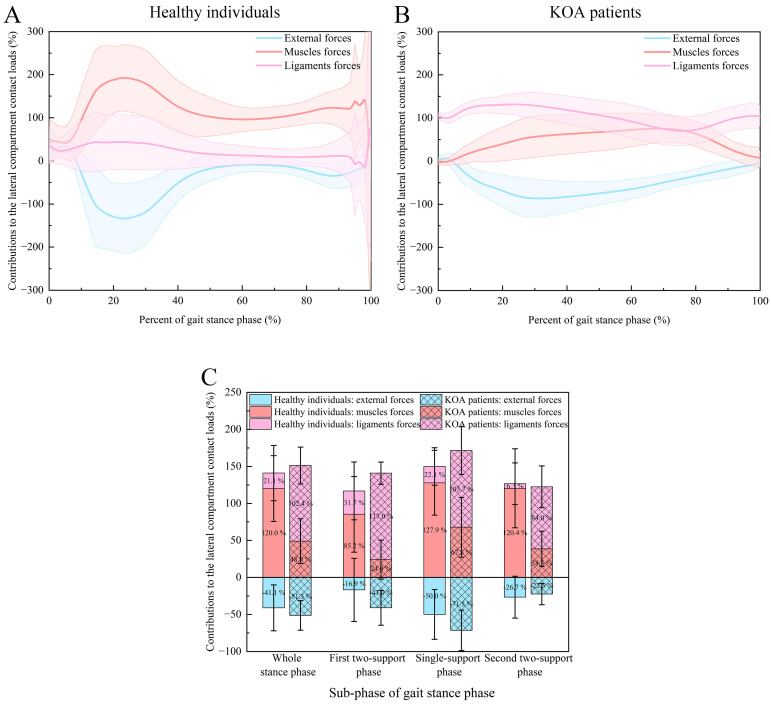
The contributions of external, muscle, and ligament forces to the lateral compartment contact loads: (**A**) contribution–time curves (mean: solid line, standard deviation: shaded area) for the healthy individuals (normalized from 0% to 100% of the gait stance phase); (**B**) contribution–time curves (mean: solid line, standard deviation: shaded area) for the KOA patients (normalized from 0% to 100% of the gait stance phase); (**C**) normalized contributions for the healthy individuals and the KOA patients during the whole stance phase, the first two-support phase, the single-support phase, and the second two-support phase.

The contributions of external, muscle, and ligament forces to the lateral compartment contact loads during the stance phase of the gait cycle are shown in Figure 4 and Table 4. For both the healthy individuals and the KOA patients, the external forces were the negative contributor to the lateral compartment contact loads; that is to say, the external forces tended to unload the lateral compartment, for both the whole stance phase (−41.1 ± 31.0% and −51.3 ± 20.0%), the first two-support phase (−16.9 ± 42.6% and −41.0 ± 23.6%), the single-support phase (−50.0 ± 33.6% and −71.5 ± 27.3%) and the second two-support phase (−26.7 ± 28.3% and −22.5 ± 14.4%). Additionally, the negative contribution of external forces during the single-support phase was significantly greater than that during the two-support phases (*p* < 0.001). Compared to the healthy individuals, the KOA patients possessed a larger negative contribution of external forces and tended to unload the lateral compartment more for both the whole stance phase and the sub-stance phases (*p* < 0.01). For the healthy individuals, the lateral compartment contact loads were contributed predominantly by the muscle forces (whole stance phase: 120.0 ± 44.6%; first two-support phase: 85.2 ± 51.2%; single-support phase: 127.9 ± 43.9%; second two-support phase: 120.4 ± 53.5%) followed by the ligament forces (whole stance phase:21.1 ± 37.3%; first two-support phase: 31.7 ± 39.2%; single-support phase: 22.1 ± 25.3%; second two-support phase: 6.3 ± 28.1%) to counteract the unloading effect of the external forces (*p* < 0.001). For both the contributions of muscle and ligament forces, there were no significant differences between the single-support and two-support phases (*p* > 0.05). However, for the KOA patients, the major and the secondary contributors have been changed as ligaments and muscle forces, respectively, for both the whole stance phase (102.4 ± 24.9% and 48.9 ± 30.2%), the first two-support phase (117.0 ± 15.0% and 24.0 ± 26.3%), the single-support phase (103.7 ± 32.3% and 67.8 ± 40.6%) and the second two-support phase (84.0 ± 28.2% and 38.5 ± 23.9%) (*p* < 0.001). Compared to the two-support phases, the contributions of muscle forces were significantly increased, and the contributions of ligament forces were significantly decreased during the singe-support phase (*p* < 0.05).

## 4. Discussion

The contributions of external, muscle, and ligament forces to the tibiofemoral compartment contact loads during gait and the differences in the relative contributions between KOA patients and healthy individuals have critical clinical implications for further improving the effect of the current biomechanical treatments of KOA, such as knee brace, wedge insole, gait modification, weight loss, muscle strength training, kinesio taping, etc. For this aim, the gait data collection experiment based on motion capture and force plate system and gait simulation analyses based on an improved musculoskeletal model with knee ligaments in twenty KOA patients and twenty healthy individuals were performed in this study. To the best of our knowledge, this was the first to quantify and compare the differences in the relative contributions of external, muscle, and ligament forces to the tibiofemoral contact loads during the gait between the KOA patient and the healthy individuals.

For the total compartment contact loads, the contribution of ligament forces was greater than 18.7% for the healthy individuals and 35.3% for the KOA patients, which was consistent with our hypothesis that the knee ligaments provided important contributions. Additionally, this study observed that the primary contributor was muscle forces for the healthy individuals, but ligament forces for the KOA patients, which was concordant with our hypothesis that the KOA would alter the relative contributions. This difference could be explained by two reasons mainly. Firstly, the quadriceps femoris and gastrocnemius muscles were the primary contributing muscles to resist the EKAM during gait [20,33,34]. Compared to the healthy individuals, the weakness of the two muscles was observed in the patients with KOA [35,36]. Therefore, the contribution of muscle forces was decreased in the KOA patients. Marriott et al. [10] pointed out that increasing muscle strength could transfer the loads from more vulnerable joint structures to muscles. We recommended that the strength training targeting the quadriceps and gastrocnemius muscles should be prioritized for the KOA patients. In addition, we advised that the knee orthotic devices should not only consider the applied orthopedic moments but also provide supplemental strength or strength training for the weaker quadriceps and gastrocnemius muscles. Secondly, the posterolateral corner, including LCL and PFL, provided the primary contribution to resisting the EKAM [20,34]. The knee varus would increase the passive ligament forces of the posterolateral corner [20,22,34] and then increase the contributions of ligament forces for the KOA patients. We suggested that the KOA patients could perform targeted stretching exercises to strengthen their knee ligaments or utilize knee orthotic devices to support their ligaments during movement, improving knee joint stability. Consistent with the previous studies [17,20,33], the greater contribution of muscle forces to the total compartment contact loads than that of the external forces was also found in this study. There were two possible reasons. One reason might be that the external forces tended to unload the lateral compartment, which might lead to a decrease in its contribution to the total compartment. Another reason might be that the contribution of the quadriceps femoris and gastrocnemius muscle forces were formed by relatively larger muscle forces rather than the moment arm, which resulted in almost symmetrically contribution to the medial and lateral compartments [20,34]. This symmetrical contribution might increase its contribution to the total compartment. Our results also showed that the contribution of external forces was greater, and the contribution of ligament forces was smaller during the single-support phase than during the two-support phases. The reasons might be twofold. First, the knee joint supported more proportion of body weight during the single-support phase than during the two-support phases. Second, the smaller ligament contribution might be due to the smaller knee varus angle during the single-support phase than that during the two-support phase [22].

For the medial compartment contact loads, external forces were the major contributors for both the healthy individuals and the KOA patients, which were in good agreement with the previous studies [18,19,20]. The reason might be that the external loads were mainly borne by the medial compartment under the influence of EKAM [22,37]. Our findings also indicated that the KOA patients had greater external forces contribution, which might be due to the greater EKAM [22]. Consistent with our hypothesis, our results showed that the ligament forces provided a contribution similar to muscle forces for healthy individuals and a negative contribution for KOA patients. Unlike our study, Shelburne et al. [20] reported that the muscle forces contributed more than the ligament forces to the medial compartment contact loads for healthy individuals. This inconsistency might be due to the differences in the muscle strength of the recruited subjects. Five healthy people aged 26 ± 3 years were recruited in the study of Shelburne et al. [20], while twenty healthy people aged 64.8 ± 5.4 years were recruited in our study. The weaker muscle strength might increase the ligament’s contributions to stabilizing the tibiofemoral joint against the external forces. The negative contribution of ligament forces for the KOA patients could be explained as follows. Due to the KOA-induced varus deformity, the strain of knee medial ligaments decreased while the strain of knee lateral ligaments increased. This might cause the direction of total knee ligament forces to shift from the medial to the lateral compartment. For the KOA patients, this shift aimed to counteract the excessive EKAM and stabilize the whole knee joint. We recommended that the targeted stretching exercises, which strengthened the knee ligaments, or the knee orthotic devices, which supported the knee ligaments, may be beneficial for repairing the negative contribution. This negative contribution led to the muscle forces not only resisting the external forces but also resisting the imbalance of ligament forces. This might be why our results found that the KOA patients had greater muscle force contributions. Our findings also indicated that the external forces provided a greater contribution, while the muscle and ligament forces provided a smaller contribution during the single-support phase than the two-support phases. These differences could be explained by three reasons mainly. Firstly, the results might be due to the greater load bearing and greater EKAM during the single-support phase [22]. Secondly, the smaller knee varus angle might reduce the contributions of ligament forces during the single-support phase [22]. Thirdly, the muscle contribution was predominantly provided by the quadriceps femoris muscle during the first two-support stance and the gastrocnemius muscle during the second two-support stance [17,19,20,33]. During the single-support phase, our study also found that the muscle forces were the primary contribution and the ligament forces were the secondary contribution for healthy individuals, while the muscle forces and ligament forces contributed equally for the KOA patients. The reason might be that the increased ligament forces contribution could have supplemented a portion of the reduced muscle forces contribution due to weaker muscle strength for the KOA patients. We suggested that the targeted interventions of the KOA patients should consider strengthening the ligaments and muscles simultaneously.

For the lateral compartment contact loads, the negative contribution of external forces was observed for both the KOA patients and the healthy individuals. The negative contribution of external forces was also found in the studies of Winby et al. [17] and Shelburne et al. [20]. The reason for the negative contribution was that the external forces tended to unload the lateral compartment. Our finding showed that the negative contribution of external forces was greater for KOA patients than for healthy individuals. We suspected the reasons might be due to the larger EKAM for the KOA patient [22]. Additionally, we also found that the negative contribution was greater during the single-support phase than during the two-support phases. The results might be due to the greater load bearing of body weight and greater EKAM during the single-support phase [22]. Consistent with our hypothesis, the lateral compartment contact loads were contributed predominantly by the muscle forces and followed by the ligament forces for the healthy individuals, and their contributions were no significant differences between single-support and two-support phases. For the KOA patients, the lateral compartment contact loads were contributed predominantly by the ligament forces and followed by the muscle forces. Compared to the two-support phases, the contributions of muscle forces were significantly increased, and the contributions of ligament forces were significantly decreased during the singe-support phase. These results may be due to the weaker muscle strength and greater passive posterolateral corner (i.e., LCL and PFL) ligament forces for the KOA patients than the healthy individuals [20,33,34,35,36].

Several potential limitations of this study should be acknowledged. Firstly, the same mechanical parameters were assumed for KOA and healthy ligaments. The ligament and muscle attachment sites were based on the cadaver data and scaled to participants based on the related attached bones in this study. For the ligament mechanical properties, Peter et al. [38] studied the effect of KOA on the mechanical properties of ACL, PCL, MCL, and LCL from human cadaveric knee joints. They found that there were negative correlations between KOA grades and ACL yield stress and between strain and LCL failure stress. However, no statistically significant correlations existed between KOA grade and MCL and PCL tensile mechanical properties. Piao et al. [39] compared the LCL tensile mechanical properties between healthy and KOA samples using rat models. The results showed that the maximum load, maximum stress, maximum displacement, and maximum strain all decreased after KOA. Jin et al. [40] compared the MCL tensile mechanical properties of healthy and KOA rabbit models. They found that the KOA reduced the maximum strain, maximum stress, maximum displacement, and elasticity modulus of MCL. Nawata et al. [41] investigated the effect of KOA on the tensile properties of rat ACL and found that the failure load and stiffness were significantly reduced compared to healthy rat ACL. For the ligament and muscle attachment sites, the cadaver data-based approach allowed for a large simplification of the modeling process but could certainly reduce the accuracy of the model. The attachment sites were mainly related to the direction of muscle and ligament forces. We acknowledged that the non-patient-specific mechanical properties and attachment sites might lead to potential errors in the computed ligament forces and then in the contributions of external, muscle, and ligament forces. In future work, the patient-specific mechanical properties and attachment sites based on MRI and ultrasound will be considered. Secondly, the individual muscle force in this study was predicted by using the inverse dynamic with muscle recruitment and the quasi-static approach, which were the traditional and validated method in commonly used musculoskeletal modeling software, such as AnyBody Modeling Software [42] and OpenSim Modeling Software (SimTK, Stanford, CA, USA) [43]. Compared to the actual pattern of muscle activation, this method had some simplifications. For example, the co-contraction of muscles was neglected. Although EMG single was used to verify the predicted muscle force in some previous studies [17,27], EMG single and muscle force were two different physical phenomena with complex related mechanisms. Thus, comparing predicted muscle forces with participant’s actual EMG signals could not be regarded as the gold standard in verifying the predicted muscle force. More reliable muscle force verification methods and EMG-driven musculoskeletal modeling simulations will be considered in future work. As a fast and efficient approach, the quasi-static approach could resolve the related biomechanical parameters at each time frame. However, the quasi-static approach did not integrate the equations of motion between the time frames, so the effect between the time frames was ignored in this method. In future work, more advanced approaches will be explored and adopted. Thirdly, only the contributions of total muscle forces and total ligament forces and only the tibiofemoral contact loads acting along the longitudinal axis of the tibia were analyzed in this study. The contributions of individual muscle and individual ligaments and the tibiofemoral contact loads acting along the other two axes were not explored. In future work, we will group the ligaments and muscles that have the same function and investigate the contributions of each ligament group and muscle group to tibiofemoral contact loads acting along all three axes. Finally, this study only compared the contributions between the healthy individuals and the patients with medial KL 3–4 KOA. The patients with KL1–2 KOA or lateral KOA were not considered in this study. We will expand recruitment in future studies to cover other KOA severities and compartments.

## 5. Conclusions

In this study, the contributions of external, muscle, and ligament forces to the tibiofemoral compartment contact load during normal gait in patients with medial KL 3–4 KOA and healthy individuals were investigated based on an improved musculoskeletal model with knee ligaments. According to the experimental and simulation results, the main conclusions can be summarized as follows:

(1) The total or lateral compartment contact loads were contributed predominantly by the muscle forces for the healthy individuals but by ligament forces for the KOA patients. The medial compartment contact loads were predominantly contributed by the external forces for the healthy individuals and the KOA patients, with greater contributions in the KOA patients.

(2) The contribution of muscle forces to the total compartment contact loads was greater than that of the external forces for both the healthy individuals and the KOA patients.

(3) The ligament forces provide a contribution similar to muscle forces to the medial compartment contact loads for healthy individuals and a negative contribution in the KOA patients.

(4) The external forces provide a negative contribution to unloading the lateral compartment in both the healthy individuals and the KOA patients, with a greater negative contribution in the KOA patients.

In conclusion, the knee ligaments provide important contributions to the tibiofemoral contact loads. Significant differences were found between the KOA patients and the healthy individuals in the relative contributions of external, muscle, and ligament forces to the tibiofemoral contact loads on the total, medial, and lateral compartments. This study has significant clinical implications for further improving the effects of the current biomechanical treatments of KOA.

## Figures and Tables

**Table 1:** Demographic information for participants.

	KOA Patients Group (*n* = 20)	Healthy Control Group (*n* = 20)	*p*
Age, years	66.2 ± 7.7	64.8 ± 5.4	0.510
Height, meters	1.56 ± 0.06	1.58 ± 0.05	0.259
Body mass, kg	66.8 ± 8.7	65.7 ± 4.6	0.621
BMI ^1^, kg/m^2^	27.6 ± 3.2	26.3 ± 2.1	0.138
Sex, male/female	4/16	6/14	0.715

^1^ BMI: body mass index.

**Table 2:** The contributions of external, muscle, and ligament forces to the total compartment contact loads during the stance phase of the gait cycle.

Phase	Type of Forces	KOA Patients Group	Healthy Control Group
Whole stance phase	External forces	24.8 ± 4.9%	31.2 ± 3.3%
	Muscle forces	32.1 ± 9.3%	46.2 ± 4.9%
	Ligament forces	43.1 ± 12.4%	22.6 ± 5.7%
	*p* values	**<0.001** ^a^; **<0.001** ^b^; **<0.001** ^c^	**<0.001** ^a^; **<0.001** ^b^; **<0.001** ^c^
First two-support stance phase	External forces	25.2 ± 6.4%	31.4 ± 5.3%
	Muscle forces	20.9 ± 7.0%	36.3 ± 8.1%
	Ligament forces	53.9 ± 11.4%	32.3 ± 8.9%
	*p* values	**0.0191** ^a^; **<0.001** ^b^; **<0.001** ^c^	**<0.001** ^a^; 0.601 ^b^; **<0.001** ^c^
Single-support stance phase	External forces	29.5 ± 5.4%	33.6 ± 4.0%
	Muscle forces	35.4 ± 12.0%	47.7 ± 5.3%
	Ligament forces	35.1 ± 14.7%	18.7 ± 5.5%
	*p* values	**<0.001** ^a^; **<0.001** ^b^; 0.934 ^c^	**<0.001** ^a^; **<0.001** ^b^; **<0.001** ^c^
Second two-support stance phase	External forces	14.6 ± 3.3%	21.3 ± 4.3%
	Muscle forces	38.3 ± 10.8%	49.7 ± 5.9%
	Ligament forces	47.1 ± 12.3%	29.0 ± 6.8%
	*p* values	**<0.001** ^a^; **<0.001** ^b^; **<0.001** ^c^	**<0.001** ^a^; **<0.001** ^b^; **<0.001** ^c^

^a^ *p* values between external and muscle forces; ^b^ *p* values between external and ligament forces; ^c^ *p* values between muscle and ligament forces. *p* values in bold indicate significant differences.

**Table 3:** The contributions of external, muscle, and ligament forces to the medial compartment contact loads during the stance phase of the gait cycle.

Phase	Type of Forces	KOA Patients Group	Healthy Control Group
Whole stance phase	External forces	79.2 ± 9.3%	61.4 ± 12.1%
	Muscle forces	35.0 ± 13.4%	19.2 ± 16.1%
	Ligament forces	−14.2 ± 13.4%	19.4 ± 22.3%
	*p* values	**<0.001** ^a^; **<0.001** ^b^; **<0.001** ^c^	**<0.001** ^a^; **<0.001** ^b^; 0.747 ^c^
First two-support stance phase	External forces	78.5 ± 12.0%	42.1 ± 15.2%
	Muscle forces	35.1 ± 16.8%	27.6 ± 24.6%
	Ligament forces	−13.6 ± 14.3%	30.3 ± 30.2%
	*p* values	**<0.001** ^a^; **<0.001** ^b^; **<0.001** ^c^	**<0.001** ^a^; **<0.001** ^b^; 0.563 ^c^
Single-support stance phase	External forces	91.2 ± 9.7%	70.0 ± 12.0%
	Muscle forces	17.0 ± 9.2%	13.5 ± 12.6%
	Ligament forces	−8.2 ± 7.4%	16.5 ± 17.7%
	*p* values	**<0.001** ^a^; **<0.001** ^b^; **<0.001** ^c^	**<0.001** ^a^; **<0.001** ^b^; 0.415 ^c^
Second two-support stance phase	External forces	53.5 ± 21.2%	49.6 ± 18.6%
	Muscle forces	75.4 ± 38.7%	28.6 ± 36.7%
	Ligament forces	−28.9 ± 42.6%	21.8 ± 22.0%
	*p* values	**<0.001** ^a^; **<0.001** ^b^; **<0.001** ^c^	**<0.001** ^a^; **<0.001** ^b^; 0.467 ^c^

^a^ *p* values between external and muscle forces; ^b^ *p* values between external and ligament forces; ^c^ *p* values between muscle and ligament forces. *p* values in bold indicate significant differences.

**Table 4:** The contributions of external, muscle, and ligament forces to the lateral compartment contact loads during the stance phase of the gait cycle.

Phase	Type of Forces	KOA Patients Group	Healthy Control Group
Whole stance phase	External forces	−51.3 ± 20.0%	−41.1 ± 31.0%
	Muscle forces	48.9 ± 30.2%	120.0 ± 44.6%
	Ligament forces	102.4 ± 24.9%	21.1 ± 37.3%
	*p* values	**<0.001** ^a^; **<0.001** ^b^; **<0.001** ^c^	**<0.001** ^a^; **<0.001** ^b^; **<0.001** ^c^
First two-support stance phase	External forces	−41.0 ± 23.6%	−16.9 ± 42.6%
	Muscle forces	24.0 ± 26.3%	85.2 ± 51.2%
	Ligament forces	117.0 ± 15.0%	31.7 ± 39.2%
	*p* values	**<0.001** ^a^; **<0.001** ^b^; **<0.001** ^c^	**0.001** ^a^; **<0.001** ^b^; **<0.001** ^c^
Single-support stance phase	External forces	−71.5 ± 27.3%	−50.0 ± 33.6%
	Muscle forces	67.8 ± 40.6%	127.9 ± 43.9%
	Ligament forces	103.7 ± 32.3%	22.1 ± 25.3%
	*p* values	**<0.001** ^a^; **<0.001** ^b^; **<0.001** ^c^	**<0.001** ^a^; **<0.001** ^b^; **<0.001** ^c^
Second two-support stance phase	External forces	−22.5 ± 14.4%	−26.7 ± 28.3%
	Muscle forces	38.5 ± 23.9%	120.4 ± 53.5%
	Ligament forces	84.0 ± 28.2%	6.3 ± 28.1%
	*p* values	**<0.001** ^a^; **<0.001** ^b^; **<0.001** ^c^	**<0.001** ^a^; **<0.001** ^b^; **<0.001** ^c^

^a^ *p* values between external and muscle forces; ^b^ *p* values between external and ligament forces; ^c^ *p* values between muscles and ligament forces. *p* values in bold indicate significant differences.

## Data Availability

All data used in this study are available from the corresponding author on a reasonable request.
